# A Meta-Analysis of the Effects of Dietary Yeast Mannan-Rich Fraction on Broiler Performance and the Implication for Greenhouse Gas Emissions from Chicken Production

**DOI:** 10.3390/ani14111595

**Published:** 2024-05-28

**Authors:** Saheed A. Salami, Jules Taylor-Pickard, Stephen A. Ross, Colm A. Moran

**Affiliations:** 1 Alltech Biotechnology Centre, Summerhill Road, A86 X006 Dunboyne, Ireland; sayodeji14@yahoo.com; 2 Solutions Deployment Team, Alltech (UK) Ltd., Ryhall Road, Stamford PE9 1TZ, UK; jpickard@alltech.com; 3 Alltech E-CO_2_, Ryhall Road, Stamford PE9 1TZ, UK; sross@alltech.com; 4 Regulatory Affairs Department, Alltech SARL, Rue Charles Amand, 14500 Vire, France

**Keywords:** yeast mannan-rich fraction, postbiotics, gut health, broiler, greenhouse gas emissions

## Abstract

**Simple Summary:**

Mannan-rich fraction (MRF) is a natural extract of *Saccharomyces cerevisiae* and can be classified into the emerging functional category of products termed postbiotics. Dietary supplementation with MRF has been extensively investigated in poultry as a natural alternative to in-feed antibiotics due to its efficacy in improving immune response, nutrient digestion, and absorption. This present study provides a comprehensive meta-analytic review of the effect of dietary MRF on broiler performance parameters and carbon footprint (CFP) of chicken production via a life cycle assessment model. The results showed that the supplementation of broiler diets with MRF improved growth performance parameters and reduced mortality. Dietary MRF had an equivalent effect to in-feed antibiotics on broiler performance, suggesting that MRF can be used in broiler nutrition as a natural alternative to in-feed antibiotics. Additionally, feeding MRF lowered the environmental impact by reducing feed and total emission intensities, on average, by −2.4% and −2.1%, respectively, contributing to sustainable chicken production.

**Abstract:**

Dietary supplementation of yeast-derived mannan-rich fraction (MRF) could improve the gastrointestinal health and production efficiency of broilers, and, consequently, lower the environmental impacts of chicken production. The objective of this meta-analysis was to quantify the retrospective effects of feeding MRF (Actigen^®^, Alltech Inc., Nicholasville, KY) on the production performance of broilers. The meta-analysis database included 27 studies and consisted of 66 comparisons of MRF-supplemented diets vs. basal (i.e., negative control) and antibiotic-supplemented (i.e., positive control) diets. A total of 34,596 broilers were involved in the comparisons and the average final age of the birds was 35 days. Additionally, the impact of feeding MRF on the carbon footprint (feed and total emission intensities) of chicken production was evaluated using the meta-analysis results of broiler performance (MRF vs. basal diets) to develop a scenario simulation that was analyzed by a life cycle assessment (LCA) model. A database of all trials (MRF vs. basal and antibiotic diets) indicated that feeding MRF increased (*p* < 0.01) average daily feed intake (ADFI; +3.7%), final body weight (FBW; +3.5%), and average daily gain (ADG; 4.1%) and improved (*p* < 0.01) feed conversion ratio (FCR; −1.7%) without affecting (*p* > 0.05) mortality. A subdatabase of MRF vs. basal diets indicated that dietary MRF increased ADFI (+4.5%), FBW (+4.7%), and ADG (+6.3%) and improved FCR (−2.2%) and mortality (−21.1%). For the subdatabase of MRF vs. antibiotic diets, both treatments exhibited equivalent effects (*p* > 0.05) on broiler performance parameters, suggesting that MRF could be an effective alternative to in-feed antibiotics. Subgroup analysis revealed that different study factors (year of study, breed/strain, production challenges, and MRF feeding duration) influenced the effect of dietary MRF on broiler performance. Simulated life cycle analysis (LCA) indicated that feeding MRF decreased feed and total emission intensities, on average, by −2.4% and −2.1%, respectively. In conclusion, these results demonstrate that dietary MRF is an effective nutritional solution for improving broiler performance, an effective alternative to in-feed antibiotic growth promoters, and reduces the environmental impact of poultry meat production.

## 1. Introduction

The continuous increase in the global demand for animal protein has intensified the production of poultry meat, with an evolving change in breeding efficiency and management practices accompanied by an increased susceptibility to production challenges. These production challenges and stressors could perturb the gut microbiome, morphology, and function, which, in turn, could impair animal health and prevent birds from achieving their genetic potential for optimum production performance. Maintaining optimal gut health is crucial for improving digestive efficiency and attenuating the negative impacts of production challenges on poultry performance and health [1]. Historically, in-feed antibiotics have been utilized at subtherapeutic doses to improve gut function and health, prevent diseases, and enhance the growth performance of chickens [2]. However, the increasing threat of zoonotic transmission of antimicrobial resistance has heightened the global restriction on the subtherapeutic use of in-feed antibiotics in livestock production [3,4]. These restrictions have stimulated further interest in the search for viable antibiotic alternatives to maintain or improve gut health and the performance efficiency of broilers [2,5].

Mannan-rich fractions (MRFs) are a natural extract of the yeast cell wall of *Saccharomyces cerevisiae* [6]. An MRF can be classified into the emerging functional category of products termed postbiotics—“a preparation of inanimate microorganisms and/or their components that confers a health benefit on the host” [7]. Dietary MRF has been extensively studied in poultry nutrition to improve the intestinal health and functions of broilers which could enhance feed digestion and nutrient utilization, immune response, and production performance [8,9]. These beneficial responses have been attributed to the effects of dietary MRF to prevent pathogen colonization in the gut, enhance intestinal morphology, favorably modulate the gut microbiota and microbial metabolism, and stimulate immune development and function [10,11,12,13,14,15,16]. Despite the considerable positive effects of dietary MRF on broiler performance, some studies have presented inconsistent results [10,17,18]. Additionally, there is variation among studies in the magnitude of broiler performance response to dietary MRF. These inconsistencies could be attributed to differences in experimental conditions, diet formulation, and health status of the birds [19]. Thus, a critical synthesis of the scientific literature is required to obtain an evidence-based conclusion regarding the efficacy of MRF supplementation in improving broiler performance.

Classical qualitative literature reviews result in subjective conclusions and do not account for possible experimental variables that could influence the effects of a nutritional intervention on animal performance [20]. In contrast, a meta-analysis is a rigorous statistical approach with greater analytical power to obtain quantitative inference about a set of response variables derived from the combination of multiple independent studies [20,21]. Moreover, a meta-analysis allows for a systematic exploration of factors that contribute to variation in the response variables, thereby providing the opportunity for wider practical knowledge on the application of nutritional interventions [21,22]. In previous meta-analytic studies, Hooge and Connolly [23] and Hooge et al. [24] utilized 9 and 18 research reports, respectively, to summarize and quantify the effect of MRF (Actigen^®^, Alltech Inc., Nicholasville, KY, USA) on broiler performance. Both meta-analyses demonstrated that supplementing basal diets (i.e., negative control diets) with MRF improved the final body weight (FBW; +3.3% to +5.4%) and improved the feed conversion ratio (FCR; −1.8% to −2.5%) and mortality (−12.5%) of broilers. Moreover, the supplementation with MRF resulted in equivalent effects on broiler performance (FBW, FCR, and mortality) compared to diets supplemented with in-feed antibiotics (i.e., positive control diets) [23,24]. However, an updated meta-analysis is essential to include the dataset from recent research trials and to elucidate the major sources of variation affecting broiler performance response to dietary MRF. Moreover, previous meta-analysis studies did not analyze the effect of dietary MRF on the average daily feed intake (ADFI) and average daily gain (ADG) of broilers. The outcomes of these performance variables (ADFI and ADG) are required for conducting the environmental impact simulation of a feeding intervention in a poultry production system [25,26].

In recent times, the environmental impacts of livestock production systems have attracted significant interest, especially due to the associated impact of greenhouse gas (GHG) emissions on climate change. Poultry products are considered to be more ecofriendly because of their lower carbon footprint (CFP, aggregated GHG emissions per unit product) compared to other livestock products [27]. Nonetheless, increasing consumer pressure and stricter climate policies have intensified the need for the poultry sector to be more environmentally sustainable [28]. Feed production and utilization is a significant source of GHG emissions, contributing 70 to 80% of the CFP of chicken production [25,26,29,30]. Several studies have demonstrated that dietary interventions that improve the feed utilization and production performance of broilers could reduce the CFP of chicken production [31,32,33]. Life cycle assessment (LCA) is an environmental accounting framework that can be used to quantify the CFP of a livestock product from the cradle-to-farm gate [34]. Recently, the Livestock Environmental Assessment and Performance Partnership (LEAP) of the Food and Agriculture Organization (FAO) published the guidelines to standardize and harmonize the assessments of the environmental performance of feed additives in the global livestock supply chain [35]. The LEAP guidelines highlight the adoption of meta-analysis results as a secondary data source for the wider substantiation of the efficacy of a feed additive, which can be subsequently applied to scenario analysis in an LCA model [35].

Therefore, the present study hypothesized that feeding MRF would improve the production efficiency of broilers and, consequently, lower the environmental impacts of chicken production. The first objective of this study was to provide an updated and comprehensive meta-analytic review of the effect of dietary MRF (Actigen^®^) on broiler performance parameters, including ADFI, FBW, ADG, FCR, and mortality. The second objective was to evaluate the impact of feeding MRF on the CFP of chicken production by conducting a scenario simulation in an LCA model using the meta-analysis results of broiler performance.

## 2. Materials and Methods

### 2.1. Search Strategy and Article Selection

**Figure 1:**
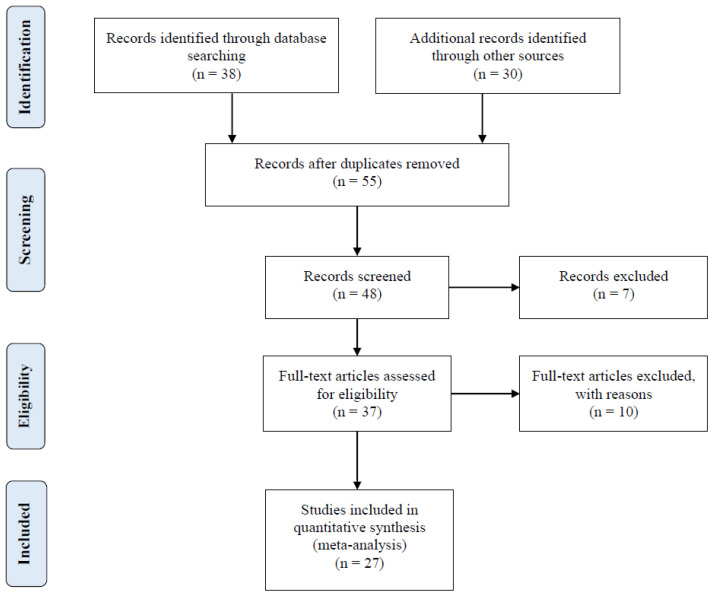
Schematics of the article search strategy and selection according to PRISMA.

A digital search was conducted through bibliographic databases (Google Scholar, Scopus, PubMed, CAB Direct, Web of Science, and Mendeley) to retrieve peer-reviewed articles evaluating the effect of a commercial MRF product (Actigen^®^, Alltech Inc., Nicholasville, KY. USA) on broiler performance. The digital search strategy was based on the inclusion of the following keywords: “broilers”, “poultry”, “mannan oligosaccharide”, “mannan-rich fraction”, “Actigen”, “growth performance”, and “broiler performance”. The literature search was conducted in 2023, and there was no prior date restriction imposed on the literature search to cover the entire duration that the MRF product had been investigated in broilers. Additionally, the company’s internal bibliographic database was searched to retrieve unpublished trial reports presented in a PhD dissertation or international scientific conferences. Figure 1 shows the schematics of the literature search and article selection applied in this meta-analysis according to the Preferred Reporting Items for Systematic Reviews and Meta-Analyses (PRISMA) Statement [36,37,38].

The literature search resulted in the initial retrieval of 68 research articles. The retrieved articles were screened and subjected to the following inclusion criteria: (1) the experiment was reported in English; (2) the experiment contained at least one negative control (i.e., basal diet) and/or positive control (i.e., antibiotic-supplemented diet), and a diet supplemented with Actigen^®^ as the MRF product; (3) the trial was conducted in broilers and adequate randomization of birds into treatments was reported; (4) the MRF dosage application rate and feeding duration were reported; (5) information describing the study factors of the experiments were provided or available upon request from the authors; and (6) information of one or more performance parameters (ADFI, FBW, ADG, FCR, and mortality) was reported or available upon request from authors. After the screening, 27 articles were selected for inclusion in the meta-analysis. Information about the articles included in the meta-analysis is summarized in Table 1.

### 2.2. Data Extraction

A database was developed by extracting data from the 27 selected articles. The database consists of 66 comparisons of MRF-supplemented diets vs. basal and antibiotic-supplemented diets. Information describing the different study factors was extracted, and the study factors include study location, year of study, breed/strain, number of experimental replicates, number of birds, MRF dosage application rate, production challenges, and MRF feeding duration (i.e., birds’ age) across the studies. The MRF dosage rates varied depending on the growth phase of the birds (Table 1), and this was largely within the manufacturer’s recommended inclusion levels. The average final age of the birds was 35 days across the studies. Furthermore, data were extracted for production performance parameters (ADFI, FBW, ADG, FCR, and mortality). The standard deviation (SD) was recorded as the measure of the variance. If the SD was not reported, it was calculated by multiplying the reported standard error (SE) of the means by the square root of the sample size [39]. Most of the studies reported a pooled SE or SD, and these estimates were used for the control and MRF treatments. However, a few trial reports provided a separate estimate of the SD or SE for each group, and these were recorded as such. The average SD from other studies was imputed for a few performance variables that did not report the measure of the variance. Following this approach, Furukawa et al. [40] and Philbrook et al. [41] provided empirical evidence supporting the accuracy of the meta-analysis results when a few missing variance data were imputed with the reported variance data from another meta-analysis or other studies in the same meta-analysis.

**Table 1:** Summary of the studies used to evaluate the effects of dietary yeast-derived mannan-rich fraction (MRF) on the production performance of broiler chickens.

Reference	Study Location	Breed/Strain	No. of Replicates	Birds per Replicate	Production Challenge	Antibiotic Treatment	MRF Dosage (kg/ton)	Duration (Day)	Response Variables
Adli and Sjofjan [17]	Indonesia	Arbor Acres	4	187	None	None	0.8 ^1^	35	ADFI, FBW, ADG, FCR, MORT
Culver et al. [42]	United Kingdom	Ross 308	8	9	None	None	0.8/0.4/0.2 ^3^	40	FBW, ADG, FCR
Fomentini et al. [43]	Brazil	Cobb 500	8	30	None	Avilamycin, halomycin	0.4/0.2/0.2 ^4^	49	ADFI, FBW, ADG, FCR
Gernat [44]	Honduras	Arbor Acres x Ross	11	57	None	Bacitracin	0.4/0.4 ^5^; 0.4/0.2 ^5^; 0.2/0.2 ^5^	42	FBW, FCR, MORT
Laustsen and Nollet [45]	Denmark	-	4	60	None	None	0.8/0.25 ^6^	34	ADG, FCR
Mathis and Brennan [46]	USA	Cobb 500	12	50	None	Bacitracin	0.8/0.4/0.2 ^7^	42	FBW, FCR
M’Sadeq, Wu, Choct, Forder and Swick [18]	Australia	Ross 308	6	10	Necrotic enteritis	Bacitracin, salinomycin	0.8/0.4/0.2 ^8^	35	ADFI, ADG, FCR, MORT
Sultan et al. [47]	Pakistan	Cobb 500	4	15	None	None	0.8 ^1^; 1.0 ^1^	21	ADFI, ADG, FCR
Brennan et al. [48]	USA	Cobb 500	12	50	None	Bacitracin, salinomycin	0.8/0.4/0.2 ^7^	42	ADFI, ADG, FCR
Corrigan, Fay, Corcionivoschi, and Murphy [10]	United Kingdom	Ross 308	3	41	Natural *Campylobacter*	None	1.3/1.0/0.6 ^9^	35	ADFI, FBW, ADG, FCR
Good [49]	USA	Cobb 500	6	6	None	None	0.4 ^1^	21	ADFI, FBW, FCR
Haese et al. [50]	Brazil	Cobb 500	8	24	Heat stress and poor litter quality	Halquinol, avilamycin	0.4/0.4 ^5^; 0.4/0.2 ^5^; 0.2/0.2 ^5^	42	ADFI, ADG, FCR, MORT
Bentea et al. [51]	Romania	Ross 308	25	1	None	None	0.8/0.4/0.2 ^2^	42	ADFI, FBW, ADG, FCR
Ivkovic et al. [52]	Serbia	Ross 308	8	38	None	None	0.2 ^1^; 0.4 ^1^; 0.8 ^1^	42	FBW, ADG, FCR, MORT
Ivkovic et al. [53]	Serbia	Ross 308	8	38	None	None	0.2 ^1^; 0.4 ^1^	42	FBW, ADG, FCR, MORT
Kay et al. [54]	United Kingdom	Ross 308	8	23	None	None	0.8/0.4/0.2 ^10^	40	ADFI, FBW, ADG, FCR
Laustsen and Nollet [55]	Denmark	—	6	60	None	None	0.8/0.5/0.3 ^11^	34	FBW, ADG
Lea, Spring, Taylor-Pickard, and Burton [19]	United Kingdom	Ross 308	12	5	None	None	0.2 ^1^; 0.4 ^1^; 0.8 ^1^	42	FBW, ADG, FCR
Macalintal et al. [56]	USA	Cobb 500	10	7	Injected LPS	None	0.4 ^1^	21	ADFI, ADG, FCR
Macalintal et al. [57]	USA	Cobb 500	10	8	Injected dexamethasone	None	0.4 ^1^	21	ADFI, ADG, FCR
Mathis [58]	USA	Cobb 500	13	50	None	Bacitracin	0.8/0.4/0.2 ^7^	42	FBW, FCR
Mathis et al. [59]	USA	Cobb 500	12	45	Poor litter quality	Bacitracin, virginiamycin	0.4 ^1^	52	FBW, FCR, MORT
Munyaka et al. [60]	Canada	Ross 308	6	60	None	Monensin, bacitracin	0.2/0.1/0.05 ^12^	42	ADFI, ADG, FCR, MORT
Nollet and Beeks [61]	Netherlands	Cobb 500	8	30	None	None	0.4 ^1^	32	FBW, ADG, FCR
Pescatore et al. [62]	USA	Cobb 500	10	6	None	None	0.4 ^1^	21	ADFI, FBW
Saleem et al. [63]	Pakistan	Hubbard	4	8	None	None	0.2 ^1^	35	ADFI, FBW, FCR
Waqas et al. [64]	Pakistan	Cobb 500	6	15	None	None	0.2 ^1^; 0.4 ^1^; 0.6 ^1^	35	ADFI, FBW, ADG, FCR, MORT

LPS: lipopolysaccharide; ADFI: average daily feed intake; FBW: final body weight; ADG: average daily gain; FCR: feed conversion ratio; MORT: mortality. ^1^ An MRF single dosage was used from the beginning until the end of the trial. ^2^ An MRF step-down dosage was used in the starter phase (1–14 days)/grower phase (15–35 days)/finisher phase (36–42 days), respectively. ^3^ An MRF step-down dosage was used in the starter phase (1–10 days)/grower phase (11–35 days)/finisher phase (36–42 days), respectively. ^4^ An MRF step-down dosage was used in the starter phase (1–21 days)/grower phase (22–42 days)/finisher phase (43–49 days), respectively. ^5^ An MRF step-down dosage was used in the starter and grower phases (1–21 days)/finisher phase (22–42 days), respectively. ^6^ An MRF step-down dosage was used in the starter (1–11 days)/grower–finisher phases (12–34 days), respectively. ^7^ An MRF step-down dosage was used in the starter phase (1–7 days)/grower phase (8–21 days)/finisher phase (22–42 days), respectively. ^8^ An MRF step-down dosage was used in the starter phase (1–10 days)/grower phase (11–24 days)/finisher phase (25–35 days), respectively. ^9^ An MRF step-down dosage was used in the starter phase (1–10 days)/grower phase (11–25 days)/finisher phase (26–35 days), respectively. ^10^ An MRF step-down dosage was used in the starter phase (1–12 days)/grower phase (13–24 days)/finisher-withdrawal phase (25–40 days), respectively. ^11^ An MRF step-down dosage was used in the starter phase (1–7 days)/grower phase (8–28 days)/finisher phase (29–34 days), respectively. ^12^ An MRF step-down dosage was used in the starter phase (1–14 days)/grower phase (15–21 days)/finisher phase (22–42 days), respectively.

### 2.3. Data Synthesis and Statistical Analysis

Comprehensive meta-analysis software (version 3, Biostat Inc., Englewood, NJ, USA) was utilized to analyze the effect of treatment comparisons in a random-effects model. The effect size of dietary MRF on broiler performance parameters was estimated using the raw mean difference (RMD) and standardized mean difference (SMD) at a 95% level of confidence interval (CI). The RMD is the sum of the mean differences of the MRF treatment relative to the control treatment in individual studies, weighted by the individual variances for each study. The RMD estimates the actual effect of treatment in unit measures. On the other hand, the SMD is the mean difference between the MRF treatment and the control treatment, which is standardized based on the SD of the MRF and control groups, resulting in a numerical dimensionless value. Cohen [65] proposed an interpretation of the SMD values as follows: 0.2, 0.5, and 0.8 are equivalent to small, medium, and large effects, respectively. Lean et al. [66] suggested that the SMD is a more robust effect size estimate when there is heterogeneity in the dataset. The effect size estimates (i.e., RMD and SMD) of all trial comparisons (MRF diets vs. basal and antibiotic diets) on performance variables were declared significant when *p* ≤ 0.05. Furthermore, subdatabases were created to perform the meta-analysis of the subgroups, evaluating how study factors/covariates influence the response of production performance variables to dietary MRF. The subdatabases were stratified into groups/subgroups based on the following study factors: treatment comparisons (MRF vs. basal diets or MRF vs. antibiotic diets), years of study (2010–2014 or 2015–2020), breed/strain (Ross or Cobb), production challenge (none or yes), and MRF feeding duration (≤day 28 or >day 28 of age). As recommended by Kelley and Kelley [67], subgroups with less than 5 study comparisons were excluded from the meta-analysis. This is the reason why only one subgroup was included in the meta-analysis of the production challenges (none) in FBW and MRF feeding duration (>day 28) in mortality.

The variability among studies (i.e., heterogeneity of effect size) was evaluated using the *I*^2^ statistic and the associated significance level of the chi-squared statistic [68]. The *I^2^* statistic was computed to describe the percentage of the total variation across studies due to heterogeneity rather than chance. The *I*^2^ values of <25%, 25 to 50%, and >50% are indicative of low, moderate, and high heterogeneity, respectively [69]. The presence of a publication bias in this meta-analysis was evaluated using the funnel plot method (funnel plot asymmetry is indicative of publication bias) [70]. Additionally, the publication bias was assessed statistically using Egger’s regression method between the SMD and SE [71], and the bias was declared significant when *p* < 0.05.

### 2.4. Life Cycle Assessment

#### 2.4.1. Goal and System Boundary

**Figure 2:**
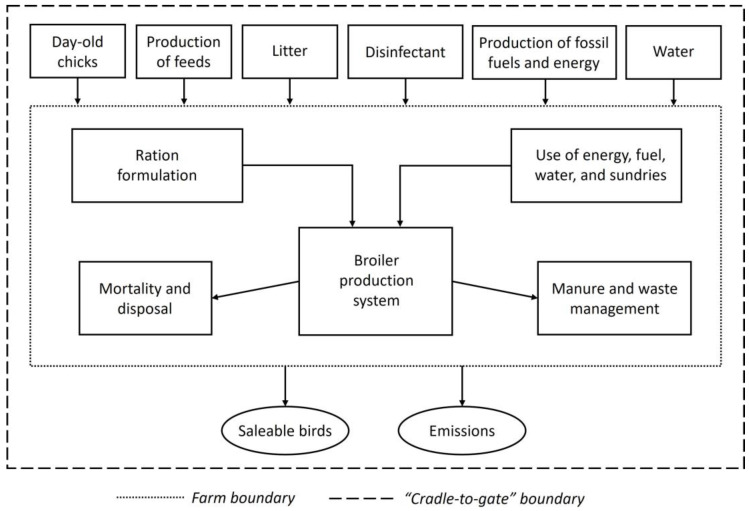
Flow chart of the main components and system boundary of broiler production evaluated in the life cycle assessment.

An LCA simulation modeling was conducted to assess the impact of feeding MRF on the CFP of broiler chicken production. The system boundary included all processes involved in broiler production up to the farm gate (“cradle-to-farm gate”). This covers all stages from the extraction or acquisition of raw materials, through the supply chain and on-farm processes, up to the point at which the birds were ready to leave the farm. Therefore, this study did not account for emissions attributed to subsequent downstream processing, packaging, or transport of chicken meat beyond the farm gate. This cradle-to-farm gate approach was consistent with the LCA methodology applied for analyzing broiler production systems in previous studies [26,72,73]. Figure 2 describes the system boundary of the LCA model analyzed in this study. The outputs from the system are saleable birds and litter. All litter was exported from the farm, and a carbon credit was allocated through a system expansion; thus, the birds were the only true product of the system. The emission intensities are presented using the following three functional units: kg CO_2_-eq/bird; kg CO_2_-eq/kg liveweight [LW]; and kg CO_2_-eq/kg carcass weight [CW] leaving the farm gate. The period of each assessment scenario was the time to reach a finishing weight of 2.5 kg, and all scenarios started with a flock of 100,000 birds placed on the farm.

#### 2.4.2. Production System and Scenarios

The production systems considered in this study were based on a model of an average conventional European-housed broiler system. The study modeled specifics of four production scenarios within this system, comprising a baseline (i.e., a scenario without MRF supplementation) and an intervention (MRF) scenario (i.e., a scenario with dietary MRF supplementation) managed on each of two diets defined by different inclusion rates of soybean meal (SBM) (i.e., low-SBM and high-SBM diets, respectively). Low- and high-SBM diets were adopted from the “realistic” sunflower diet and baseline (soya) diet, respectively, as presented in Leinonen, Williams, Waller, and Kyriazakis [25] (Supplementary Table S1). The MRF scenario was defined by the supplementary inclusion of Actigen^®^ in the diets, while the feed ingredient inclusions in the ration formulations were consistent in the baseline and MRF scenarios. All feeds were purchased and imported onto the broiler farm, therefore there were no system inputs or emissions (e.g., inorganic fertilizer, direct and indirect N_2_O losses from field applications, soils, and crop residues) associated with the cultivation and harvest of home-grown crops. Day-old chicks were supplied to the farm by an independent hatchery and placed in a flock starting with 100,000 birds. The hatchery phase was assumed identical for all scenarios, and the subsequent growing period lasted 46 days in the baseline scenario. Birds in all scenarios were managed on three successive formulated rations (defined as starter, grower, and finisher diets) following common industry practice.

#### 2.4.3. Inventory Analysis

Information on production system parameters was obtained from the average of over 450 commercial European broiler farm environmental assessments conducted by Alltech E-CO_2_ in the same system type from 2017 to 2020 (Alltech E-CO_2_, Stamford, UK). Chicks were assumed to have a body mass of 56 g upon placement in the flock, and the baseline mortality was 5.2%. Finished birds had, on average, a 2.5 kg body mass at the end of the growing period, with a kill-out of 71% consistent across all four scenarios. Data on the production characteristics and performance of birds in the baseline and MRF scenarios are presented in Supplementary Table S2. Compared to the baseline, the data used for the MRF scenario were determined by the relative improvement percentage in the production performance observed through the meta-analysis results. The four ration formulations contained the same feed ingredients, and specific information relating to the low-SBM and high-SBM diets are presented in Supplementary Table S1. Average commercial data were also employed to account for general resource use, including electricity, fossil fuels, disinfectant, litter, and water use for consumption by the flock and washdown. It was assumed in this study that all litter was stored on-farm until the end of the flock period, whereupon it was exported to be used as an organic fertilizer. All viable birds were sold at the end of the production period, and dead birds were sent to a rendering plant.

#### 2.4.4. Impact Assessment

The environmental impact assessment was conducted using Alltech E-CO_2_’s Poultry EA™ (broiler) model (Alltech E-CO_2_, Stamford, UK), a bespoke CFP calculator employed commercially in the broiler industry and independently accredited by the Carbon Trust according to the British Standards Institute’s publicly available specification 2050:2011 (PAS:2050) [74] and the International Standard ISO 14067:2018 [75]. The model was designed following the Intergovernmental Panel on Climate Change (IPCC) guidelines for tier 2 methodology [76] for livestock emissions, with the ability to implement tier 3 data when available for inputs such as feed intake and dietary crude protein content. Nitrogen excreted by birds was determined from the total feed intake per bird, the weighted protein content of the feed, and the estimated percentage of dietary nitrogen excreted by the animal, following IPCC [76]. Direct and indirect emissions of N_2_O and CH_4_ arising from manure and litter management were estimated using tier 2 equations from the IPCC [76].

Embedded emissions associated with the cultivation, production, and delivery of purchased feeds were estimated using data sourced from FeedPrint software, developed by Wageningen University and Research, the Netherlands [77]. Feed data were assumed to be directly compatible with the methodology employed in this study, as the process level data were also stated to be compliant with PAS:2050 and co-products of crop production were treated based on economic allocation. Feeds were assumed to be of typical European market production, and SBM was assumed to be of South American origin, including the associated emission burden from land-use change.

Embedded emissions in chicks arriving on the farm were estimated to be 0.56 kg CO_2_-eq per bird, as retrieved from the Alltech E-CO_2_’s database. Coefficients used for transport emissions associated with the delivery of chicks, purchased feeds, and removal of dead birds were sourced from DEFRA [78]. Additionally, emission factors employed for the production and use of energy and fossil fuels were obtained from DEFRA [78]. A system expansion process was applied to estimate a carbon offset for the fertilizer value of exported litter, estimated by employing the nutrient content and availabilities recommended by DEFRA [79] and the coefficients for the avoided production of inorganic fertilizers as described by Hoxha and Christensen [80].

Emissions (kg CO_2_-eq) for major GHG were calculated using conversion factors for a 100-year time horizon, defined to be 28 and 265 times for CH_4_ and N_2_O, respectively, as consistent with reporting under the 5th Assessment Report of the IPCC (AR5) [81]. Feed emission intensity (i.e., GHG emissions attributed to the feed use per functional unit) and total emission intensity (i.e., total GHG emissions per functional unit) were subsequently estimated for each of the four scenarios for the production life cycle of 100,000 birds placed in the broiler system.

## 3. Results

### 3.1. Study Characteristics

A brief description of the studies included in this meta-analysis is presented in Table 1. The 27 included studies were conducted across 12 countries (8 from the United States; 4 from the United Kingdom; 3 from Pakistan; 2 each from Brazil, Denmark, and Serbia; 1 each from Canada, Honduras, Australia, Indonesia, the Netherlands, and Romania) from 2010 to 2023 (Table 1). The dataset consists of 66 dietary comparisons of MRF vs. basal and antibiotic diets, comprising 69.7% and 30.3% comparisons of MRF vs. basal diets and MRF vs. antibiotic diets, respectively. A total of 34,596 birds were involved in the comparisons, and the average final age of the birds was 35 days across all studies. The MRF dosage rate was applied in 51.9% of the studies using a step-down inclusion approach, whereas 48.1% of the studies applied single MRF doses throughout the trial. In the step-down studies, the predominant MRF dosage rate was 0.8/0.4/0.2 kg/ton used in the starter/grower/finisher phases, respectively, while the average MRF dosage in the single-dose studies was 0.5 kg/ton.

The summary statistics of broiler performance variables (ADFI, FBW, FCR, and mortality) evaluated in this meta-analysis are shown in Table 2. The averages observed for the performance variables were ADFI (93.95 g/d), FBW (2262.99 g), ADG (59.54 g/d), FCR (1.67), and mortality (7.63%). There were considerable variations in ADFI (37.81 to 140.36 g/d), FBW (475.32 to 3261.00 g), ADG (27.42 to 92.93 g/d), FCR (1.27 to 2.02), and mortality (1.12 to 48.30%). In general, the global coverage of the dataset provides a good representation to derive significant conclusions from this meta-analysis while acknowledging the diversity in different study factors that could account for the variation in the dataset.

### 3.2. Average Daily Feed Intake

**Figure 3:**
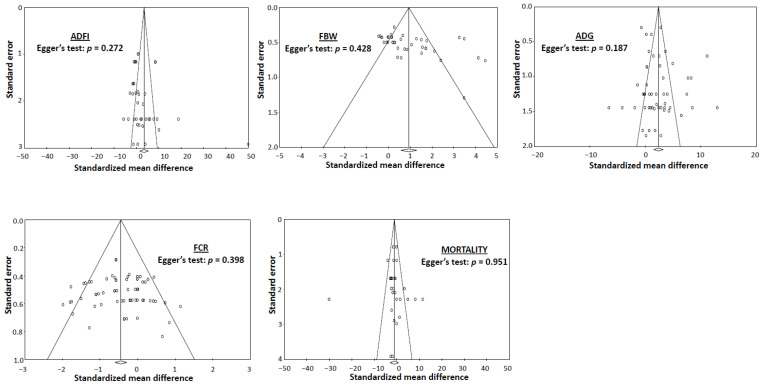
Funnel plots and the significance (*p*-value) of the Egger’s test in assessing the publication bias of the dataset analyzed for average daily feed intake (ADFI), final body weight (FBW), average daily gain (ADG), feed conversion ratio (FCR), and mortality. Open circles indicate individual study comparisons included in the meta-analysis.

The results of the ADFI response to MRF supplementation are presented in Table 3. The pooled effect of all trials (MRF vs. basal and antibiotic diets) indicated that feeding MRF relatively increased (*p* < 0.01) ADFI by 3.7% (RMD = +3.45 g/d, CI: 1.50 to 5.40). This significant effect was mainly due to the comparison of MRF vs. basal diets (RMD = +3.82 g/d, CI: 1.87 to 5.77), whereas no effect (*p* > 0.05) was found when MRF vs. antibiotic diets was compared. Significant heterogeneity was found in the ADFI dataset, and the subgroup analysis revealed that different study factors could influence ADFI response to dietary MRF (Table 3). Dietary MRF did not influence ADFI in studies conducted from 2010 to 2014 but increased ADFI (RMD = +3.30 g/d, CI: 1.37 to 5.23) in later studies conducted from 2015 to 2020. Broiler breed/strain has a marked effect on ADFI response to dietary MRF, with Ross broilers showing an increased ADFI (RMD = +4.43 g/d, CI: 1.97 to 6.90), but no effect was found with Cobb broilers. Dietary MRF increased (*p* < 0.05) ADFI (RMD = +2.05 g/d, CI: 0.61 to 3.50) in studies with normal production conditions and tended (*p* = 0.09) to increase ADFI (RMD = +3.20 g/d, CI: −0.48 to +6.88) in studies involving production challenges. Moreover, there was an increase (*p* < 0.05) in ADFI regardless of the MRF feeding duration (≤day 28 or >day 28 of age). Overall, the subgroup analysis of the effect of study factors on the ADFI yielded significant heterogeneity in all comparisons, except when the dataset was analyzed for studies conducted from 2010 to 2014 and studies involving Cobb broilers. However, the symmetrical shape of the funnel plot, and the result of the Egger’s test indicated that there was no significant publication bias in the studies used for the meta-analysis of the ADFI (Figure 3).

### 3.3. Final Body Weight

As shown in Table 4, dietary MRF increased (*p* < 0.01) FBW in all trials (MRF diets vs. basal and antibiotic diets) by 3.5% (RMD = +78.17 g, CI: 44.61 to 111.74) and this positive outcome was predominantly driven by the effect of MRF vs. basal diets (RMD = +99.78 g, CI: 60.45 to 139.10). In contrast, the comparison of MRF vs. antibiotic diets displayed a similar effect (*p* > 0.05) on FBW. Subgroup analysis indicated that dietary MRF increased (*p* < 0.05) FBW within an RMD range of 50.92 and 140.14 g regardless of the year of study (2010 to 2014 or 2015 to 2020), broiler breed/strain (Ross or Cobb), production challenge (none), and MRF feeding duration (≤day 28 or >day 28 of age) (Table 4). The subgroup analysis did not eliminate the significant heterogeneity levels (*I*^2^ > 50%) except when the dataset was analyzed for subgroup MRF vs. antibiotic diets. Nonetheless, there was no evidence of significant publication bias in the studies used for the meta-analysis of FBW response (Figure 3).

### 3.4. Average Daily Gain

Supplementation of MRF enhanced (*p* < 0.01) ADG in all trials (MRF diets vs. basal and antibiotic diets) by 4.1% (RMD = +2.40 g/d, CI: 1.54 to 3.25) and this effect was primarily due to increased ADG when MRF vs. basal diets was compared (RMD = +3.46 g/d, CI: 2.27 to 4.44) (Table 5). The comparison of MRF vs. antibiotic diets yielded an equivalent effect (*p* > 0.05) on ADG. Dietary MRF increased (*p* < 0.01) ADG within an RMD range of 1.63 and 2.98 g/d regardless of the year of study (2010 to 2014 or 2015 to 2020) and broiler breed/strain (Ross or Cobb). Supplementation of MRF positively influenced (*p* < 0.01) ADG (RMD = +2.49 g/d, CI: 1.61 to 3.38) in studies with normal production conditions but no effect was found (*p* > 0.05) in studies involving production challenges. Additionally, dietary MRF increased ADG (*p* < 0.01) when fed for >28 days of age (RMD = +2.53 g/d, CI: 1.65 to 3.40). Regarding birds fed MRF for ≤day 28 of age, the RMD showed that MRF only tended (*p* = 0.07) to increase ADG (RMD = +2.03 g/d, CI: −0.13 to +4.18) whereas SMD indicated that MRF significantly increased (*p* = 0.021) ADG with a small to large magnitude of response (SMD = 0.90; CI: 0.14 to 1.66). High heterogeneity levels (*I*^2^ > 50%) were observed in all study factor comparisons evaluated by the subgroup analysis (Table 5) but significant publication bias was not detected in the studies included in the meta-analysis of ADG (Figure 3).

### 3.5. Feed Conversion Ratio

The pooled effect in all trials (MRF vs. basal and antibiotic diets) indicated that feeding MRF relatively decreased FCR by −1.7% (RMD = −0.028, CI: −0.037 to −0.019; Table 6). The response effect of dietary MRF in decreasing pooled FCR was mainly apparent (*p* < 0.01) when MRF vs. basal diets (RMD = −0.038, CI: −0.049 to −0.027) was compared but no effect (*p* > 0.05) was found for the comparison of MRF vs. antibiotic diets (Table 6). Effect size estimates (RMD and SMD) of subgroup analysis revealed that dietary MRF consistently reduced (*p* < 0.05) FCR under the following different study factors: year of study (2010 to 2014 or 2015 to 2020), broiler breed/strain (Ross or Cobb), production challenge (none) and MRF feeding duration (≤day 28 or >day 28 of age). Moderate but significant heterogeneity exists in the FCR dataset; however, insignificant heterogeneity was observed in the subgroup analysis of MRF vs. antibiotic diets, production challenge (yes) and MRF feeding duration (≤day 28 of age) (Table 6). No evidence of publication bias was found in the FCR meta-analysis as shown by the funnel plot and Egger’s test (Figure 3).

### 3.6. Mortality

The effect size estimates (RMD and SMD) of all trials (MRF vs. basal and antibiotic diets) showed that supplemental MRF did not influence mortality (Table 7). However, treatment subgrouping of the mortality dataset indicated that dietary MRF relatively reduced (*p* = 0.02) mortality by 21.1% (RMD = −1.10, CI: −2.05 to −0.16) when MRF vs. basal diets were compared, and no effect was found (*p* > 0.05) for the comparison of the MRF vs. antibiotic diets. Similarly, dietary MRF elicited a significant effect (*p* < 0.05) in reducing mortality for studies conducted from 2015 to 2020 (RMD = −1.00, CI: −1.89 to −0.12) and studies involving normal production conditions (RMD = −1.28, CI: −2.10 to −0.45). However, effect size estimates of other subgroups showed numerical reductions in mortality (*p* > 0.05). The overall mortality dataset indicated a high and significant heterogeneity level (*I*^2^ = 61.39%; Table 7) but no evidence of significant publication bias was found (Figure 3). Moreover, subgroups of MRF vs. basal diets, year of study (2015 to 2020), broiler breed/strain (Cobb), and production challenge (none) eliminated the significant heterogeneity in the dataset.

### 3.7. Feed and Total Emission Intensities

The impact of feeding MRF (MRF vs. basal diets) on the environmental footprint of chicken production was evaluated using feed and total emission intensities as CFP metrics expressed in three functional units (emissions per bird, emissions per LW, and emissions per CW) under two diet scenarios (low-SBM and high-SBM diets; Table 8). A summary detailing the estimated emissions from contributing categories within the life cycle assessment of the baseline and MRF scenarios is presented in Supplementary Table S3. The simulated LCA results show that feed emission intensity accounts for, on average, 75.2 and 76.8% of the total emission intensity in the low- and high-SBM diets, respectively. Regardless of the functional unit, feed and total emission intensities were lower in the low-SBM diet scenarios, on average, by −9.0% and −7.0%, respectively, compared to the high-SBM diet scenarios. For the three functional units, dietary MRF decreased the feed emission intensity of low- and high-SBM diets, on average, by −2.45 and −2.41%, respectively. Feeding MRF to broilers reduced total emission intensity in the low- and high-SBM diet scenarios when expressed as emissions per bird (−0.13 and −0.14 kg CO_2_-eq/bird), emissions per LW (−0.05 and −0.05 kg CO_2_-eq/kg LW), and emissions per CW (−0.08 and −0.08 kg CO_2_-eq/kg CW), respectively. These reductions in total emission intensity imply that feeding MRF lowers the environmental footprint of chicken production, on average, by −2.14 and −2.05% in the low- and high-SBM diets, respectively. Overall, feeding MRF decreased feed and total emission intensities, on average, by −2.4% and −2.1%, respectively, across all four production/dietary scenarios.

## 4. Discussion

Gut health encompasses the maintenance of homeostasis and functionality between intestinal microbiota, morphology, immunity, and nutrient digestion [82,83]. Birds differ in their susceptibility to impaired gut health due to variations in genetics, feed composition and quality, gastrointestinal microbiota, metabolic adaptation, stress, and infections. Nutritional strategies that improve gut health could play a pivotal role in enhancing production efficiency, animal welfare, food safety, and environmental sustainability of poultry production [1,82]. It is well documented that dietary MRF improves the gut health and performance of broilers under diverse production conditions [8,9]. In the present study, a meta-analysis was conducted to quantify the retrospective effects of feeding MRF (Actigen^®^) on the production performance of broilers. Consequently, the meta-analysis results of broiler performance improvements were utilized to develop an LCA scenario simulation to assess the impact of feeding MRF on the environmental footprint of chicken production.

The pooled analyses of all trials (MRF vs. basal and antibiotic diets) demonstrated that feeding MRF improved ADFI, FBW, ADG, and FCR by +3.7%, +3.5%, +4.1%, and −1.7%, respectively. Further stratification of the treatment comparisons indicated that supplementing MRF in a basal diet (MRF vs. basal diets) improved ADFI, FBW, ADG, FCR, and mortality by +4.5%, +4.7%, +6.3%, −2.2% and −21.1%, respectively. However, the comparison of the dietary MRF and antibiotics (MRF vs. antibiotic diets) exhibited equivalent effects on broiler performance parameters, suggesting that MRF could be an effective alternative to in-feed antibiotics. The present results are consistent with those reported in two previous meta-analyses, in which supplementing basal diets with MRF increased the FBW (+3.3% to +5.4%) and decreased the FCR (−1.8% to −2.5%) and mortality (−12.5%) of broilers [23,24]. Moreover, these previous meta-analysis studies corroborate our results that dietary MRF exerted similar effects on broiler performance (FBW, FCR, and mortality) compared to in-feed antibiotics.

It is noteworthy that the present study is the first to quantitatively enumerate the pooled effect of dietary MRF on ADFI and ADG. The stimulatory effect of dietary MRF on ADFI could be indirectly related to its effects in optimizing intestinal health and function, which facilitates efficient feed digestion and better nutrient utilization and absorption [8,84]. The positive relationship between feed intake and growth rate [85] could explain why increased ADFI resulted in higher ADG and FBW in birds fed supplemental MRF. Moreover, the effect of dietary MRF in preventing pathogen colonization and improving gut health could elicit an energy-sparing benefit in birds by maximizing the utilization of dietary energy and valuable nutrients for production and growth instead of partitioning nutrients toward immune response mechanisms [86]. Consequently, improved feed efficiency, as shown with the lower FCRs, implies that dietary MRF reduced the amount of feed required per kg of broiler weight gain by 28 g (all trial comparisons) and 38 g (MRF vs. basal diets), respectively. This positive effect can be crucial for increasing profitability, since feed accounts for approximately 70% of broiler production costs [87]. Moreover, the wide range of FCR values (1.27–2.02) found across the studies included in this meta-analysis are consistent with those previously reported for commercial broiler strains (1.24–2.50) [88,89], which reflect considerable variation in the FCR of broiler genotypes at different age and production systems.

Furthermore, increasing consumer demand for sustainable, ethical, and antibiotic-free animal protein has been identified as one of the major challenges confronting the future viability of the poultry industry [90]. In practice, mortality may represent a significant economic loss in poultry production and is often used as a key metric for assessing bird health and welfare. Mortality can be exacerbated when birds are exposed to pathogenic infections and stressors, and in-feed antibiotics have been historically used to improve broiler performance, mitigate disease incidence, and lower mortality rates [2]. However, the subtherapeutic use of in-feed antibiotics has been progressively prohibited globally due to the elevated risk of antibiotic resistance and the consequent threat to the treatment of bacterial infections in animals and humans [4]. The present results demonstrate that supplementing MRF in basal broiler diets was effective in reducing mortality and exhibited similar efficacy as in-feed antibiotics in lowering mortality. This suggests that dietary MRF can be effectively used as a natural alternative to in-feed antibiotics while contributing to sustainable, ethical, and antibiotic-free broiler production. The positive impacts of dietary MRF on the health and welfare of birds could be related to its ability to enhance immune modulation and reduce pathogen colonization in the gut through mutual exclusion [8,9].

A meta-analysis compares and combines the effect estimates of treatment from multiple individual studies and can also be used to explore the between-study variability or heterogeneity of the treatment effects [66]. In the current meta-analysis, high heterogeneities exist in the dataset analyzed for the performance variables, suggesting that significant differences underlie the results of the studies used in this meta-analysis. To explore the sources of these heterogeneities, a subgroup analysis was utilized to quantitatively evaluate some study factors contributing to the variation in the performance response of broilers fed dietary MRF. Additionally, the subgroup analysis can provide useful insight into the influence of study factors on the practical application of dietary MRF in broiler production. In general, feeding MRF for a short or long growth period (≤28 or >28 days of age) consistently improved the growth performance of broilers regardless of the breed/strain (Ross or Cobb) over the last decade (2010–2020). This suggests that dietary MRF has steadily maintained its efficacy in improving birds’ gut health and productivity despite progressive genetic selection and production intensification, which have increased the metabolic and physiological demands in producing better feed-efficient and fast-growing broiler lines [91]. Furthermore, the positive effects of dietary MRF on growth performance were more prominent in broilers raised under normal production conditions. Nonetheless, broilers fed MRF exhibit numerical improvements in growth performance when subjected to production challenges including bacterial infections, heat stress, poor litter quality, and induced metabolic stress. A multitude of studies have shown that production challenges such as bacterial infections [92] and heat stress [93] can compromise gut health and growth performance of broilers due to adaptive response, immune system activation, and immune-response protein synthesis [94,95]. Thus, the immunomodulatory effect of dietary MRF might have decreased the adverse effects of these production challenges on broiler performance.

Feed efficiency improvement in broilers can positively influence sustainable chicken production metrics including production costs, GHG emissions, land-use changes, water footprint, feed-to-food competition, nutrient and mineral emissions, primary energy utilization, and biodiversity [96]. In this study, we utilized an LCA model to quantify how the effect of dietary MRF on broiler performance improvements influences the CFP (feed and total emission intensities) of chicken production. Feed emission intensity is a function of the emissions per kg of feed and the feed efficiency of broilers. Our results reveal that feed emission intensity accounts for 75.2 and 76.8% of the total emission intensity of low- and high-SBM diets, respectively. This concurs with the assertion that poultry diets constitute 70 to 80% of the total GHG emissions associated with broiler production [25,26,29,30] or egg production [97,98,99]. The GHG emissions from feed production are mainly linked to fossil energy use, nitrous oxide emissions from the cultivated fields, and the impact of land-use change (LUC) resulting from the cultivation of feed crops on lands that have been recently converted from natural vegetation [98]. The SBM sourced from South America is usually associated with high LUC and is used widely in Europe and Asia as a protein source in animal feed [100,101]. This explains why replacing SBM with sunflower meal in the low-SBM diets resulted in lower feed (−9%) and total (−7%) emission intensities relative to the high-SBM diet scenarios. Indeed, this observation highlights the significance of accounting for environmental impact metrics in diet formulation and the strategic use of feed ingredients associated with low GHG emissions could reduce the CFP of chicken production [25,102].

Furthermore, our LCA modeling indicated that the total emission intensities (2.42–2.65 kg CO_2_-eq/kg LW and 3.40–3.74 kg CO_2_-eq/kg CW) found in this study were within the range of previously reported emissions per LW (1.3–4.2 kg CO_2_-eq/kg LW) [31,73,103] or emissions per CW (2.7–4.4 kg CO_2_-eq/kg CW) [26,104,105] in conventional broiler production systems. Across all dietary scenarios, feeding MRF decreased feed and total emission intensities by −2.4% and −2.1%, respectively. This reduction in simulated CFP is attributed to lower mortality and improved growth rate with a consequent decrease in days-to-market weight. Considering that dietary MRF reduced emissions per CW by −0.08 kg CO_2_-eq/kg CW, this implies that feeding MRF to produce 1000 metric tons of broiler carcass would result in emission savings of 80 tons CO_2_-eq. In perspective, this carbon emission saving is equivalent to taking 52 cars off the road in a year, or the annual electricity use in 54 houses in the UK, or 93 intercontinental return flights (per passenger) from London to New York.

## 5. Conclusions

These meta-analysis results demonstrate that supplementation of broiler diets with MRF improved growth performance, reduced mortality, and, consequently, decreased chicken production’s CFP. Moreover, dietary MRF can support the birds’ performance to an equivalent effect of in-feed antibiotics and, therefore, can be effectively used in broiler nutrition as a natural alternative to in-feed antibiotics. Feeding MRF to broilers contributes to sustainable poultry production by positively impacting productivity, economic benefit, resource efficiency, animal welfare, antibiotic reduction, and environmental stewardship.

## Figures and Tables

**Table 2:** Descriptive statistics of broiler performance variables included in the meta-analysis.

Item	*N*	Mean	Minimum	Maximum	SD
ADFI (g/d)	40	93.95	37.81	140.36	24.33
FBW (g)	39	2262.99	475.32	3261.00	604.49
ADG (g/d)	51	59.54	27.42	92.93	13.08
FCR	64	1.67	1.27	2.02	0.19
Mortality (%)	28	7.63	1.12	48.30	6.97

ADFI: average daily feed intake; FBW: final body weight; ADG: average daily gain; FCR: feed conversion ratio; *N*: number of comparisons; SD: standard deviation.

**Table 3:** Summary of the effect size estimates for the average daily feed intake (g/d) of broiler chickens supplemented with dietary yeast-derived mannan-rich fractions (MRFs).

Group/Subgroup ^1^	*N*	Control Mean (SD)	Effect Size Estimates						Heterogeneity Tests	
			RMD (95% CI)	SE	*p*-Value	SMD (95% CI)	SE	*p*-Value	*I*^2^ (%)	*p*-Value
All trials	40	92.73 (24.28)	3.45 (1.50, 5.40)	1.00	0.001	0.55 (0.21, 0.89)	0.17	0.002	74.99	<0.001
*Treatment comparisons*										
MRF vs. basal diets	22	84.49 (24.86)	3.82 (1.87, 5.77)	1.00	<0.001	0.88 (0.44, 1.33)	0.23	<0.001	73.71	<0.001
MRF vs. antibiotic diets	18	102.81 (19.83)	0.43 (−0.95, 1.81)	0.70	0.542	0.07 (−0.27, 0.41)	0.17	0.682	46.07	0.017
*Year of study*										
2010–2014	12	98.75 (27.40)	0.21 (−0.57, 1.00)	0.40	0.593	0.084 (−0.186, 0.353)	0.14	0.543	0.00	0.728
2015–2020	28	90.16 (22.86)	3.30 (1.37, 5.23)	0.98	0.001	0.73 (0.29, 1.17)	0.22	0.001	76.64	<0.001
*Breed/strain*										
Ross	19	98.24 (20.63)	4.43 (1.97, 6.90)	1.26	<0.001	0.99 (0.42, 1.57)	0.29	0.001	80.08	<0.001
Cobb	19	88.00 (27.94)	0.22 (−0.47, 0.90)	0.35	0.533	0.072 (−0.15, 0.30)	0.11	0.526	0.00	0.868
*Production challenge*										
None	28	94.53 (26.42)	2.05 (0.61, 3.50)	0.74	0.005	0.510 (0.15, 0.87)	0.19	0.006	72.11	<0.001
Yes	12	88.55 (18.70)	3.20 (−0.48, 6.88)	1.88	0.088	0.58 (−0.09, 1.25)	0.34	0.092	74.30	<0.001
*MRF feeding duration (birds’ age)*										
≤day 28	15	70.38 (20.24)	2.78 (0.50, 5.06)	1.17	0.017	0.70 (0.15, 1.26)	0.28	0.013	78.68	<0.001
>day 28	25	106.15 (14.81)	2.07 (0.47, 3.68)	0.82	0.011	0.35 (0.01, 0.68)	0.17	0.042	56.39	<0.001

*N*: number of comparisons; RMD: raw mean difference and its associated 95% confidence interval; SMD: standardized mean difference and its associated 95% confidence interval; SE: standard error. *I*^2^: percentage of variation and associated significance level (*p*-value) of chi-squared statistic. ^1^ Studies were stratified into groups and subgroups by study factors that could influence production performance.

**Table 4:** Summary of effect size estimates for final body weight (g) of broiler chickens supplemented with dietary yeast-derived mannan-rich fractions (MRFs).

Group/Subgroup ^1^	*N*	Control Mean (SD)	Effect Size Estimates						Heterogeneity Tests	
			RMD (95% CI)	SE	*p*-Value	SMD (95% CI)	SE	*p*-Value	*I*^2^ (%)	*p*-Value
All trials	39	2223.60 (598.53)	78.17 (44.61, 111.74)	17.13	<0.001	0.94 (0.57, 1.30)	0.19	<0.001	81.48	<0.001
*Treatment comparisons*										
MRF vs. basal diets	31	2105.73 (587.76)	99.78 (60.45, 139.10)	20.06	<0.001	1.22 (0.79, 1.65)	0.22	<0.001	81.70	<0.001
MRF vs. antibiotic diets	8	2680.35 (405.20)	−5.09 (−26.48, 16.30)	10.91	0.641	−0.07 (−0.37, 0.23)	0.15	0.652	0.00	0.897
*Year of study*										
2010–2014	27	2222.56 (522.16)	59.93 (25.35, 94.51)	17.64	<0.001	0.71 (0.33, 1.08)	0.19	<0.001	76.43	<0.001
2015–2020	12	2225.95 (770.13)	120.93 (52.76, 189.09)	34.78	0.001	1.47 (0.657, 2.27)	0.41	<0.001	85.37	<0.001
*Breed/strain*										
Ross	15	2136.21 (575.69)	140.14 (74.59, 205.70)	33.45	<0.001	1.75 (0.96, 2.53)	0.40	<0.001	88.66	<0.001
Cobb	15	2240.65 (772.09)	50.92 (21.81, 80.04)	14.86	0.001	0.66 (0.28, 1.04)	0.19	<0.001	56.61	0.004
*Production challenge*										
None	36	2185.49 (604.45)	77.79 (42.49, 113.09)	18.01	<0.001	0.93 (0.55, 1.31)	0.19	<0.001	81.66	<0.001
*MRF feeding duration (birds’ age)*										
≤day 28	7	1451.45 (841.68)	107.62 (18.66, 196.58)	45.39	0.018	1.48 (0.39, 2.58)	0.56	0.008	90.80	<0.001
>day 28	32	2392.51 (371.39)	71.17 (35.71, 106.63)	18.09	<0.001	0.79 (0.43, 1.14)	0.18	<0.001	74.94	<0.001

*N*: number of comparisons; RMD: raw mean difference and its associated 95% confidence interval; SMD: standardized mean difference and its associated 95% confidence interval; SE: standard error. *I*^2^: percentage of variation and associated significance level (*p*-value) of chi-squared statistic. ^1^ Studies were stratified into groups and subgroups by study factors that could influence production performance.

**Table 5:** Summary of effect size estimates for average daily gain (g/d) of broiler chickens supplemented with dietary yeast-derived mannan-rich fractions (MRFs).

Group/Subgroup ^1^	*N*	Control Mean (SD)	Effect Size Estimates						Heterogeneity Tests	
			RMD (95% CI)	SE	*p*-Value	SMD (95% CI)	SE	*p*-Value	*I*^2^ (%)	*p*-Value
All trials	51	58.35 (12.68)	2.40 (1.54, 3.25)	0.43	<0.001	0.98 (0.64, 1.33)	0.18	<0.001	79.85	<0.001
*Treatment comparisons*										
MRF vs. basal diets	33	55.18 (13.11)	3.46 (2.27, 4.44)	0.55	<0.001	1.42 (0.97, 1.87)	0.23	<0.001	81.26	<0.001
MRF vs. antibiotic diets	18	64.17 (9.69)	0.54 (−0.35, 1.41)	0.45	0.237	0.26 (−0.18, 0.70)	0.23	0.244	66.76	<0.001
*Year of study*										
2010–2014	24	59.59 (9.68)	2.40 (1.40, 3.40)	0.51	<0.001	0.99 (0.55, 1.44)	0.23	<0.001	76.00	<0.001
2015–2020	27	57.25 (14.96)	2.37 (0.90, 3.85)	0.75	0.002	0.99 (0.46, 1.53)	0.27	<0.001	82.91	<0.001
*Breed/strain*										
Ross	29	62.62 (12.21)	2.98 (1.42, 4.54)	0.79	<0.001	1.13 (0.61, 1.64)	0.26	<0.001	83.92	<0.001
Cobb	18	51.39 (11.91)	1.63 (0.77, 2.50)	0.44	<0.001	0.90 (0.40, 1.41)	0.26	<0.001	74.78	<0.001
*Production challenge*										
None	39	59.03 (12.74)	2.49 (1.61, 3.38)	0.45	<0.001	1.07 (0.71, 1.43)	0.18	<0.001	77.17	<0.001
Yes	12	56.15 (12.79)	2.09 (−0.76, 2.93)	1.45	0.151	0.74 (−0.20, 1.67)	0.48	0.122	84.93	<0.001
*MRF feeding duration (birds’ age)*										
≤day 28	15	52.64 (18.18)	2.03 (−0.13, 4.18)	1.10	0.066	0.90 (0.14, 1.66)	0.39	0.021	87.59	<0.001
>day 28	36	60.73 (8.82)	2.53 (1.65, 3.40)	0.45	<0.001	1.01 (0.64, 1.39)	0.19	<0.001	74.07	<0.001

*N*: number of comparisons; RMD: raw mean difference and its associated 95% confidence interval; SMD: standardized mean difference and its associated 95% confidence interval; SE: standard error. *I*^2^: percentage of variation and associated significance level (*p*-value) of chi-squared statistic. ^1^ Studies were stratified into groups and subgroups by study factors that could influence production performance.

**Table 6:** Summary of effect size estimates for feed conversion ratio of broiler chickens supplemented with dietary yeast-derived mannan-rich fractions (MRFs).

Group/Subgroup ^1^	*N*	Control Mean (SD)	Effect Size Estimates						Heterogeneity Tests	
			RMD (95% CI)	SE	*p*-Value	SMD (95% CI)	SE	*p*-Value	*I*^2^ (%)	*p*-Value
All trials	64	1.680 (0.203)	−0.028 (−0.037, −0.019)	0.005	<0.001	−0.454 (−0.609, −0.298)	0.079	<0.001	37.36	0.002
*Treatment comparisons*										
MRF vs. basal diets	40	1.696 (0.199)	−0.038 (−0.049, −0.027)	0.006	<0.001	−0.654 (−0.858, −0.450)	0.104	<0.001	40.21	0.005
MRF vs. antibiotic diets	24	1.655 (0.210)	−0.007 (−0.019, 0.005)	0.006	0.228	−0.133 (−0.328, 0.063)	0.100	0.183	0.00	0.727
*Year of study*										
2010–2014	36	1.788 (0.125)	−0.033 (−0.044, −0.023)	0.005	<0.001	−0.569 (−0.769, −0.368)	0.102	<0.001	38.86	0.010
2015–2020	28	1.541 (0.201)	−0.019 (−0.035, −0.002)	0.009	0.030	−0.281 (−0.523, −0.039)	0.123	0.023	32.47	0.051
*Breed/strain*										
Ross	29	1.626 (0.236)	−0.027 (−0.039, −0.014)	0.006	<0.001	−0.439 (−0.687, −0.192)	0.126	<0.001	43.84	0.007
Cobb	25	1.743 (0.171)	−0.039 (−0.056, −0.022)	0.009	<0.001	−0.589 (−0.841, −0.338)	0.128	<0.001	40.74	0.019
*Production challenge*										
None	50	1.712 (0.194)	−0.034 (−0.043, −0.025)	0.004	<0.001	−0.519 (−0.675, −0.363)	0.080	<0.001	24.12	0.067
Yes	14	1.567 (0.199)	−0.009 (−0.034, 0.015)	0.013	0.454	−0.173 (−0.617, 0.270)	0.226	0.444	59.10	0.003
*MRF feeding duration (birds’ age)*										
≤day 28	16	1.477 (0.197)	−0.022 (−0.039, −0.004)	0.009	0.014	−0.310 (−0.549, −0.071)	0.122	0.011	9.06	0.350
>day 28	48	1.748 (0.155)	−0.030 (−0.041, −0.020)	0.005	<0.001	−0.518 (−0.709, −0.327)	0.098	<0.001	42.85	0.001

*N*: number of comparisons; RMD: raw mean difference and its associated 95% confidence interval; SMD: standardized mean difference and its associated 95% confidence interval; SE: standard error. *I*^2^: percentage of variation and associated significance level (*p*-value) of chi-squared statistic. ^1^ Studies were stratified into groups and subgroups by study factors that could influence production performance.

**Table 7:** Summary of effect size estimates for mortality (%) of broiler chickens supplemented with dietary yeast-derived mannan-rich fractions (MRFs).

Group/Subgroup ^1^	*N*	Control Mean (SD)	Effect Size Estimates						Heterogeneity Tests	
			RMD (95% CI)	SE	*p*-Value	SMD (95% CI)	SE	*p*-Value	*I*^2^ (%)	*p*-Value
All trials	28	8.18 (8.41)	−1.11 (−2.99, 0.77)	0.96	0.248	−0.18 (−0.50, 0.14)	0.16	0.263	61.39	<0.001
*Treatment comparisons*										
MRF vs. basal diets	15	5.22 (1.59)	−1.10 (−2.05, −0.16)	0.48	0.023	−0.32 (−0.62, −0.03)	0.15	0.030	19.32	0.238
MRF vs. antibiotic diets	13	11.59 (11.52)	−1.50 (−5.92, 2.93)	2.26	0.508	−0.05 (−0.72, 0.62)	0.34	0.880	79.22	<0.001
*Year of study*										
2010–2014	18	10.10 (10.03)	−1.02 (−4.13, 2.09)	1.59	0.522	−0.09 (−0.59, 0.41)	0.25	0.730	74.58	<0.001
2015–2020	10	4.72 (1.20)	−1.00 (−1.89, −0.12)	0.45	0.027	−0.35 (−0.66, −0.03)	0.16	0.032	0.00	0.988
*Breed/strain*										
Ross	13	9.45 (11.80)	−1.18 (−5.64, 3.28)	2.27	0.603	−0.06 (−0.78, 0.67)	0.37	0.876	79.08	<0.001
Cobb	8	7.74 (5.17)	−0.73 (−1.70, 0.24)	0.50	0.141	−0.23 (−0.58, 0.12)	0.18	0.197	2.56	0.410
*Production challenge*										
None	20	5.61 (1.53)	−1.28 (−2.10, −0.45)	0.42	0.002	−0.31 (−0.54, −0.09)	0.12	0.007	0.00	0.529
Yes	8	14.61 (14.15)	−1.49 (−7.11, 4.14)	2.87	0.605	−0.03 (−1.10, 1.05)	0.55	0.958	86.01	<0.001
*MRF feeding duration (birds’ age)*										
>day 28	28	8.18 (8.41)	−1.11 (−2.99, 0.77)	0.96	0.248	−0.18 (−0.50, 0.14)	0.16	0.263	61.39	<0.001

*N*: number of comparisons; RMD: raw mean difference and its associated 95% confidence interval; SMD: standardized mean difference and its associated 95% confidence interval; SE: standard error. *I*^2^: percentage of variation and associated significance level (*p*-value) of chi-squared statistic. ^1^ Studies were stratified into groups and subgroups by study factors that could influence production performance.

**Table 8:** Simulated impact of supplementing dietary yeast-derived mannan-rich fraction in low- and high-soybean meal (SBM) diet scenarios on the carbon footprint of broiler production.

Category/Functional Unit	Low-SBM Diet			High-SBM Diet		
	Baseline	MRF	% Change	Baseline	MRF	% Change
*Feed emission intensity*						
Emissions per bird (kg CO_2_-eq/bird)	4.65	4.54	−2.37%	5.11	4.99	−2.35%
Emissions per live weight (kg CO_2_-eq/kg LW)	1.86	1.81	−2.69%	2.04	1.99	−2.45%
Emissions per carcass weight (kg CO_2_-eq/kg CW)	2.62	2.56	−2.29%	2.88	2.81	−2.43%
*Total emission intensity*						
Emissions per bird (kg CO_2_-eq/bird)	6.17	6.04	−2.11%	6.64	6.50	−2.11%
Emissions per live weight (kg CO_2_-eq/kg LW)	2.47	2.42	−2.02%	2.65	2.60	−1.89%
Emissions per carcass weight (kg CO_2_-eq/kg CW)	3.48	3.40	−2.30%	3.74	3.66	−2.14%

LW: live weight; CW: carcass weight.

## Data Availability

The datasets analyzed in the present study are available upon request from the authors.

## Supplementary Materials

The following supporting information can be downloaded at: https://www.mdpi.com/article/10.3390/ani14111595/s1, Table S1: Ingredient and nutrient composition of formulated broiler diets used in the life cycle assessment [106]; Table S2: Data describing production characteristics and broiler performance of baseline and dietary yeast mannan-rich fraction (MRF) scenarios; Table S3: Breakdown of emissions and production system output from the life cycle assessment of baseline and dietary yeast mannan-rich fraction (MRF) scenarios managed on low- and high-soya bean meal (SBM) diets.
