# Supplementary material for: A Meta-Analysis of the Effects of Dietary Yeast Mannan-Rich Fraction on Broiler Performance and the Implication for Greenhouse Gas Emissions from Chicken Production
Source: Animals (Basel). 2024 May 28;14(11):1595. doi: 10.3390/ani14111595 (PMC11171374; doi:10.3390/ani14111595)
Supplement: Supplementary file 1 [file animals-14-01595-s001.zip › animals-2962944-supplementary.pdf]

SUPPLEMENTARY MATERIAL

Table S1. Ingredient and nutrient composition of formulated broiler diets used in the life cycle assessment

	Starter phase (day 0 - 10)		Grower phase (day 11 - 24)		Finisher phase (day 25 - slaughter)	
Ingredient (%)	High-SBM	Low-SBM	High-SBM	Low-SBM	High-SBM	Low-SBM
Wheat	54.42	54.97	55.20	54.30	56.02	51.74
Whole wheat	-		5.00	5.00	10.00	10.00
Rapeseed whole	5.00	5.00	7.50	7.50	10.00	10.00
Soybean meal	33.50	30.00	25.50	22.00	18.00	16.00
Sunflower meal	-	2.50	-	3.75	-	5.00
Vegetable oil blend	0.70	0.50	2.00	2.40	2.50	3.50
Soya oil	2.70	3.20	1.60	1.80	0.70	1.00
Limestone	1.40	1.40	1.20	1.20	1.00	1.00
Monocalcium phosphate	0.92	0.85	0.75	0.65	0.50	0.45
Sodium bicarbonate	0.18	0.18	0.15	0.15	0.18	0.18
Sodium chloride	0.20	0.20	0.17	0.17	0.15	0.15
Lysine-HCl	0.28	0.38	0.25	0.33	0.31	0.34
DL-methionine	0.32	0.35	0.30	0.32	0.26	0.25
L-threonine	0.10	0.15	0.10	0.12	0.10	0.11
Valine	-	0.04		0.03	-	-
Enzyme	0.03	0.03	0.03	0.03	0.03	0.03
Premix	0.25	0.25	0.25	0.25	0.25	0.25
Total	100	100	100	100	100	100
Nutrient composition						
Energy (MJ/Kg)	12.7	12.7	13.1	13.1	13.4	13.4
Protein (%)	22.8	22.2	20.0	19.6	18.5	17.8
Lysine	1.44	1.41	1.20	1.20	1.08	1.08
Methionine	12.7	0.65	0.60	0.59	0.51	0.52
Calcium	1.00	1.00	0.90	0.90	0.80	0.80
Phosphorus	0.55	0.58	0.49	0.53	0.44	0.47
Available Phosphorus	0.47	0.47	0.42	0.42	0.36	0.36

Low- and high-SBM diets are adopted from the “realistic” sunflower diet and baseline (soya) diet, respectively, as presented in Leinonen et al. 2013.

Table S2. Data describing production characteristics and broiler performance of baseline and dietary yeast mannan-rich fraction (MRF) scenarios

Item	Baseline	MRF
Number of birds placed	100,000	100,000
Mortality (%)	5.2	4.1
Average daily feed intake (g/d)	84.5	88.3
Average daily gain (g/d)	55.0	58.5
Slaughter weight (kg)	2.5	2.5
Number of days to slaughter (d)	46	43
Kill-out (%)	71	71

Table S3. Breakdown of emissions and production system output from the life cycle assessment of baseline and dietary yeast mannan-rich fraction (MRF) scenarios managed on low- and high-soya bean meal (SBM) diets

	**Low-SBM Diet**		**High-SBM Diet**	
Emissions Contributing Category ($kg\ CO_2$-eq)	Baseline	MRF	Baseline	MRF
Placed chicks	56,354	56,354	56,354	56,354
Litter/bedding	10,117	10,117	10,117	10,117
Water use	1,466	1,466	1,466	1,466
Disinfectants	2,151	2,151	2,151	2,151
Starter feed	19,342	19,104	17,648	17,431
Grower feed	175,489	173,330	198,838	196,392
Finisher feed	245,642	242,620	267,657	264,364
Transport delivery of feed	4,970	4,909	4,970	4,909
Electricity	41,972	41,972	41,972	41,972
Fossil fuel	7,132	7,132	7,132	7,132
Manure management	21,560	21,295	22,180	21,907
Deadstock incineration/disposal	64	51	64	51
Exported manure	-1,324	-1,324	-1,324	-1,324
**Total emissions**	584,935	579,177	629,225	622,922
Production				
Total birds finished (n)	94,800	95,890	94,800	95,890
Total liveweight finished (kg)	237,000	239,725	237,000	239,725
Total carcass weight (kg)	168,270	170,205	168,270	170,205

References

Leinonen, I., Williams, A. G., Waller, A. H., & Kyriazakis, I. (2013). Comparing the environmental impacts of alternative protein crops in poultry diets: The consequences of uncertainty. Agricultural systems, 121, 33-42.
